# The Heat Is On: Climate Change Implications for Pregnant Women with Sickle Cell Disease

**DOI:** 10.1089/whr.2024.0146

**Published:** 2025-03-24

**Authors:** Rachelle Chanmany Pastor, Lisa Roberts, Akshat Jain, Shan Tamares

**Affiliations:** ^1^ School of Nursing, Loma Linda University, Loma Linda, California, United States.; ^2^ Sickle Cell Disease Program for Children and Young Adults, Loma Linda University, Loma Linda, California, United States.; ^3^ University Libraries, Loma Linda University, Loma Linda, California, United States.

**Keywords:** climate change, sickle cell disease, pregnancy, maternal health, health implications

## Abstract

Sickle cell disease (SCD), a serious, chronic blood disorder is the most common genetic blood disease in the United States affecting 100,000 people and disproportionately affecting the African American population. Pregnancy is particularly risky for people with SCD due to higher risk of developing pregnancy-related complications compared with people without the disease. For African American pregnant women with SCD, the risk of maternal morbidity and mortality is up to 10 times higher. Physiological changes during pregnancy increase the risk of vaso-occlusive episodes (VOEs), acute chest syndrome, venous thromboembolic events, and infections. Dehydration increases risk as it triggers sickling of red blood cells, leading to painful VOEs and further increasing the risk of aforementioned complications. Climate change, observed since the mid-20th century, is evidenced by the increasing trend of global temperature, hurricanes, floods, and heat waves. Climate changes can profoundly impact people with SCD, as elevated temperatures result in increased core body temperatures, blood hyperosmolality, and dehydration. Assisted by a research librarian, a literature search was undertaken of major databases (PubMed, Embase, and Google Scholar), with delimiters of publication between 2019 and 2024 and human subjects, and 477 studies were retrieved. After meticulous screening, 20 relevant articles were analyzed. Evidence linking climate change impact to increased risk for pregnant people with SCD is lacking. Further research is needed to examine the phenomenon and mitigate this unique risk of climate change. SCD clinical guidelines stress the importance of preventing dehydration. Clinicians play a critical role in educating this vulnerable population about risks, including dehydration and exposure to extreme heat.

## Introduction

About 100,000 people in the United States are affected by sickle cell disease (SCD)^1^ while an estimated 7.74 million people worldwide are living with the disease,^2^ specifically with high prevalence in sub-Saharan Africa, the Middle East, and India.^3^ In the United States, most people who have SCD are of African ancestry with about 1 in 13 African American babies born with sickle cell trait, while 1 in 365 African American babies are born with the disease.^4^

SCD is a lifelong, severely disabling, inherited blood disorder that causes the production of abnormal hemoglobin, denoted as sickle hemoglobin (HbS). HbS causes rigid, sickle-shaped red blood cells (RBCs) that get trapped in small blood vessels and block blood flow.^2,5,6^ The disease is characterized by microvascular occlusion, abnormal regulation of erythrocyte volume, ischemia-reperfusion injury, inflammation and oxidant damage, endothelial injury, and leukocyte and platelet activation.^5^ These conditions lead to vaso-occlusion, multiorgan dysfunction, and other significant health complications such as vaso-occlusive events (VOEs) causing pain episodes, splenic sequestration, renal dysfunction, acute chest syndrome, and stroke.^2,6,7^ Furthermore, SCD is also associated with increased susceptibility to infectious diseases, higher risk of maternal and fetal complications, and maternal mortality.^2,8,9^

### Pregnancy and sickle cell disease

Pregnancy with SCD can be precarious and challenging to manage due to the increased risk for maternal morbidity and mortality.^8–10^ Physiological changes like hormonal alteration, hemodilution, and hypercoagulability that occur during *all* pregnancies are exacerbated in pregnant women with SCD, and increase the risk of VOEs, acute chest syndrome, venous thromboembolic events, infections, and obstetrical complications, such as preeclampsia, in-utero fetal death, preterm delivery (mostly induced), and intrauterine growth restriction.^8–10^ The results of a systematic review (SR) and meta-analysis by Aghamolaei et al.^11^ on feto-maternal outcomes for 9,827 pregnant women with SCD (compared with women without SCD) showed a statistically significant increased risk of postpartum hemorrhage (RR: 3.2, 95% CI: 1.2, 8), premature delivery (RR: 3.2, 95% CI: 1.2, 8), pregnancy-induced hypertension (RR: 4.8, 95% CI: 1.6, 14.5), preeclampsia, (RR: 2.1, 95% CI: 1.5, 2.9), eclampsia (RR: 2.7, 95% CI: 1.1, 6.4), cesarean section, (RR: 1.8, 95% CI: 1.5, 2.2), and maternal death (RR: 7.2, 95% CI: 3.7, 19). These findings were consistent with the earlier results of the meta-analysis by Boafor et al.^8^ who found that SCD was associated with increased risk of preeclampsia (pooled OR 2.05, 95% CI: 1.47, 2.85), eclampsia (pooled OR 3.02, 95% CI: 1.20, 7.58), and maternal mortality (pooled OR 10.91, 95% CI: 1.83, 65.11, *p* = 0.009). The findings of both SRs echo Oteng-Ntim et al.^12^ findings from their SR and meta-analysis of 26,349 pregnant women with SCD who were at increased risk for preeclampsia (RR: 2.43; 95% CI: 1.75, 3.39), eclampsia (RR: 4.89; 95% CI: 1.97, 12.16), cesarean section (RR: 1.50; 95% CI: 1.26, 1.79), preterm delivery (RR: 2.21; 95% CI: 1.47, 3.31), and maternal mortality (RR: 5.98; 95% CI: 1.94, 18.44). Altogether, these studies strongly demonstrate the increased likelihood of maternal morbidity and mortality for pregnant women with SCD in comparison with the population of pregnant women without SCD.

Pregnancies in SCD are often complicated by VOEs,^13^ which are sudden onset, painful episodes usually lasting 5–6 days that may be generalized or affect one area of the body. The abdomen, chest, vertebrae, and extremities are common sites for VOEs.^13,14^ One major trigger of VOEs is dehydration, as insufficient fluid in the body can cause RBCs to shrink, sickle, and clump together.^15^ The presence of these dense, dehydrated RBCs is relevant, as these types of RBCs are physiologically altered, leading to vaso‐occlusion and subsequent hypoxemic organ damage.^16^ Impaired oxygen delivery to the tissues complicated by inflammation propagates a cycle of acute pain.^17^ A serious maternal complication is bone marrow embolism, called pseudotoxaemia, which is characterized by systolic hypertension and proteinuria without edema.^18^ Patients are advised to avoid all potential triggers, including stress, cold temperatures, severe fatigue, infection, and dehydration, which are important predisposing factors for VOEs.^15,17,19^

### Climate change and impact on health

For years, scientists have warned of the warming effects of climate change, often referred to as global warming. There has been an increase of 0.6°C–0.9°C from 1905 to 2005 on the Earth’s surface temperature and climate models indicate that we should expect temperatures to get even warmer, with further increases of about 2°C–6°C before the end of the 21st century.^20^ This upward trend is primarily caused by increased human emissions of heat-trapping greenhouse gases resulting in major changes in the environment, such as shrinking glaciers and ice sheets, accelerated sea level rise, and prolonged, more intense heat waves.^21^ Other climate changes such as longer periods of drought, more frequent wildfires, and extreme rainfall from tropical cyclones are occurring faster than previously predicted. Consequently, the Intergovernmental Panel on Climate Change issued a dire warning “the scientific evidence is unequivocal: climate change is a threat to human wellbeing and the health of the planet.”^21^

As global temperatures continue to rise, heat-related health burdens of disease are on an upward trend.^20,22,23^ When the environmental temperature is greater than the core body temperature, increased cardiac output, sweat production, redirection of blood flow to the skin, and skin vasodilation are the body’s compensatory responses to reduce the risk of overheating.^20,24^ These responses can be diminished in the elderly population, people on diuretic and anticholinergic drugs, or susceptible groups with chronic illness,^24,25^ such as SCD. Exposure to high ambient temperatures is a contributing factor to the exacerbation of many preexisting health conditions including cardiovascular disease, renal disease, diabetes, asthma, and mental health.^26^ The latest SR and meta-analyses on the association between high ambient temperature and heat-related illnesses by Faurie et al.^23^ included 37 studies spanning more than two decades. The researchers found that every 1°C increase in temperature was associated with an 18% increase in heat-related morbidity (RR: 1.18, 95% CI: 1.16, 1.19). The effect was greater for heat illness or heatstroke (RR: 1.45, 95% CI: 1.38, 1.53) than for dehydration or fluid and electrolyte disturbance (RR: 1.02, 95% CI: 1.02, 1.03). However, mortality from heat illness was even more significant. Mortality increased by 35% with a 1°C temperature rise (RR: 1.35, 95% CI: 1.29, 1.41). They concluded that the mortality risk was highest for people over age 65 years (RR: 1.25; 95% CI: 1.20, 1.30) and those living in subtropical climates (RR: 1.25; 95% CI: 1.21, 1.29).

### High ambient temperature and pregnant women with sickle cell disease

Climate change affects weather patterns, leading to altered temperatures, increased frequency of extreme weather events, and variations in humidity levels. High ambient temperatures may overwhelm the capacity of maternal thermoregulatory mechanisms to dissipate heat during pregnancy and labor.^27^ Pregnancy is marked by a hypercoagulable state that increases the risk of thrombosis and other complications with resulting morbidity, and in severe cases, maternal death.^28,29^ These physiological conditions compounded by climate change may have a profound impact on individuals living with SCD. Therefore, the purpose of the article is to explore what is known about these combined concerns.

## Materials and Methods

With the assistance of an academic research librarian, the following major databases were accessed: PubMed, Embase, and Google Scholar, with de-limiters of published years of 2019 through 2024 and human subjects. The search string comprised official subject headings appropriate for their respective databases, along with keywords related to the following main concepts: (“sickle cell anemia” OR “hb ss disease” OR “ss disease (sickle cell)”: “haemoglobin ss disease” OR “hemoglobin ss disease” OR “homozygous sickle cell anemia” OR “homozygous sickle cell anemia” OR “homozygous sickle cell disease”) AND (“pregnant woman” OR “pregnant women” OR “pregnant women with sickle cell disease”) AND (“dehydration” OR “fluid depletion”: OR “fluid deprivation”: OR “fluid loss” OR “loss fluid”) AND (“climate change” OR “climate sensitivity” OR “climate variability”).

**FIG. 1.**
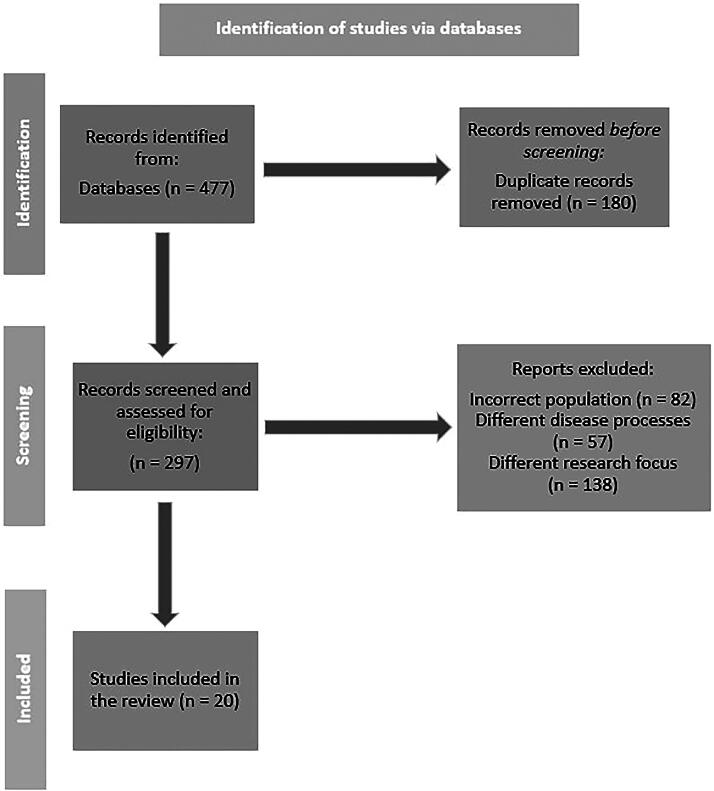
Diagram.

A total of 477 studies from the three databases were retrieved and imported to EndNote X21, a bibliographical software. EndNote was utilized to manage all the relevant studies by first eliminating duplicate studies, a total of 180 articles. Of the remaining studies, titles and abstracts were meticulously screened, and 277 articles were excluded because of incorrect population, different disease processes, or research focus. The remaining 20 relevant articles were analyzed and included in the review (see Fig. 1).

## Results

Evidence examining the phenomenon of the impact of climate change on pregnant women with SCD is lacking. There were no articles specifically exploring the correlation between climate change and women’s health outcomes in SCD, and moreover no studies examining direct effect of climate change on the SCD general population were found, despite their vulnerability. Because evidence on the phenomenon is lacking, the four concepts in the phenomenon: (1) SCD, (2) pregnant women with SCD, (3) dehydration, and (4) climate change were reviewed individually, noting some overlap. Of the studies that discussed SCD, two were SRs^2,3^ and four were review articles,^5–7,17^ while on the topic of SCD and pregnancy, there were three SRs and meta-analyses,^8,11,12^ and four review articles.^9,10,28,30^ On the topic of dehydration and pregnancy, one review article was found.^27^ On climate change, a modeling study,^31^ three review articles,^20,22,32^ and an SR and meta-analysis^23^ were included. Only one review article was found on climate change and SCD,^15^ affirming the potential impact of climate change on the prevalence, management, and outcomes of the disease. Obeagu and colleague suggested that extreme temperature fluctuations and frequent and intense heat waves related to climate change pose a significant challenge to individuals with SCD because with high ambient temperatures and dehydration, the risk for SCD-related complications is increased. From the search results, only high-quality articles and reports were selected. JBI Critical Appraisal Tools were referenced to identify the inclusion of essential elements, with an average of 82%, the quality of included reports was deemed acceptable (https://jbi.global/critical-appraisal-tools). Ultimately, the findings from this review suggest a gap in the literature regarding the phenomenon as a whole and warrant future research to investigate the possibility of a relationship between the concepts identified.

## Discussion

As ambient temperatures continue to rise due to climate change, people’s health and well-being are impacted.^20,22^ Heat waves and excessive heat are occurring more frequently and intensely placing millions of people under heat warnings,^21^ resulting in scores of heat-related illnesses and deaths.^23,31,32^ A recent modeling study by García-León et al.^31^ assessed the current and future temperature-related mortality risk from climate change across 1,368 European regions (30 countries—27 European Union member states, Norway, Switzerland, and the United Kingdom). One of the study findings revealed that heat-related deaths were six times higher in Southern Europe than in Northern Europe between 1991 and 2020 with a median of 43,729 heat-related deaths (95% CI: 39,880, 45,921). The researchers estimated that with the currently projected 3°C increase in global warming on top of the documented 3° rise from 1905 to 2005, heat-related mortality could rise to an additional 54,974 (95% CI: 24,112, 80,676) by the year 2100, mostly impacting the Southern regions of Europe.

The Fifth National Climate Assessment, a U.S. congressionally mandated interagency effort that examines the impacts, risks, and responses of climate change, reported the *profound negative effects* of climate change on human’s physical, mental, spiritual, and community health through climate-related extreme events such as extreme temperatures, droughts, wildfires, floods, and storms,^33^ The report specified that health risks from climate change include higher rates of heat-related morbidity and mortality with increases in some adverse pregnancy outcomes, disproportionately affecting underresourced communities, people of color, and people with chronic diseases. Unfortunately, this description characterizes the SCD population, indicating that they are at higher risk for heat-related adverse health effects. Health disparities persist, including inadequate access to high-quality specialized care for SCD, even in high-resource countries such as the United States.^34–36^

For pregnant women with SCD, close monitoring throughout the pregnancy stages by a multidisciplinary care team is a priority. The team needs to evaluate precipitating factors like exposure to high ambient temperatures and dehydration,^37^ as well as the individual’s access to health resources to mitigate the risk of VOEs and other complications of SCD likely to be exacerbated. An estimated 50%–70% of pregnant women with SCD are admitted to the hospital at least once, with VOEs (34%) as the most common reason for admission followed by acute chest syndrome (8%), urinary tract infection (7%), and preeclampsia (6%).^38^

Multiple studies have found that SCD is negatively associated with maternal health outcomes.^8–12^ Pregnant women with SCD compared to the healthy population are at elevated risk for developing pregnancy-related complications such as pregnancy-induced hypertension, preeclampsia, eclampsia, cesarean section, premature delivery, postpartum hemorrhage, and maternal death. Additionally, maternal complications such as severe anemia, renal disease, or pulmonary hypertension from chronic underlying organ dysfunction can be aggravated during pregnancy. These risks can be attributed to the physiological changes associated with pregnancies in women with SCD, such as increased metabolic demands, hypercoagulable state, endothelial dysfunction, severe anemia, and increased VOEs.^28^ General awareness among all stakeholders, particularly care providers in both the acute and ambulatory outpatient settings, needs to be emphasized. Regions that are experiencing high temperatures multiple months out of the year, not just in the United States, but globally (especially India and sub-Saharan Africa) need to be especially mindful of heat toxicity and its related detrimental health outcomes affecting vulnerable populations.

Managing SCD is multifaceted, involving a multidisciplinary team, close regular medical care, patient education, and avoidance of triggers of VOEs like dehydration.^5,15,30^ The latest clinical practice guidelines from the American Society of Hematology can be accessed from the link provided: https://www.hematology.org/education/clinicians/guidelines-and-quality-care/clinical-practice-guidelines/sickle-cell-disease-guidelines. However, these guidelines are not specific for pregnant women with SCD and national guidelines still need to be further developed as more patients are now surviving to reproductive age.^30^ The impact of rising ambient temperatures is a factor that should be considered in managing the condition and preventing pregnancy-related complications. Additional consideration should be given to underresourced people or those who lack access to cooling measures and/or adequate water supply, people of color, people with mental health or substance use disorders, and those experiencing homelessness. Dehydration, a heat-related illness that can occur in a hot climate,^23^ is preventable, and patients must be educated about the importance of adequate hydration and avoidance of high-temperature exposure specifically during heat waves.^10,15,30^

Consequently, this is a public health concern that merits further study to understand the implications of heat resulting from climate change on pregnant women with SCD. We sought to bring awareness to healthcare providers and family caregivers on the implications of high ambient temperature resulting from climate change, to help prevent complications and care for people with SCD who are particularly vulnerable to climate change.

### Limitation

The review was an initial assessment of the literature in examining current evidence on the phenomenon of climate change’s impact on this high-risk maternal population (women with SCD). Delimiting the search to the last 5 years might have yielded inadequate results regarding SCD; however, it is appropriate for the rapidly evolving topic of climate change.^33^ The combined concepts introduce a novel phenomenon, therefore, current literature was apropos.

## Conclusions

Evidence shows climate change’s negative impact on human health and well-being. Greater health impacts can result when multiple climate-related extreme events occur simultaneously or consecutively, that is, back-to-back heatwaves, heatwaves during wildfires, or drinking water contamination after flooding.^32,33^ Specifically for maternal health, rising temperatures and extreme climate-related events are associated with adverse pregnancy and birth outcomes.^33^ Due to the compounded risks of climate change and dehydration on pregnant women with SCD, this phenomenon needs to be further analyzed as a whole. The paucity of evidence on the phenomenon warrants further research to examine the potential relationship between the constructs, as study findings can subsequently inform and add to the clinical management of SCD among this vulnerable population.

## Abbreviations Used

HbS: Sickle Hemoglobin
JBI: Joanna Briggs Institute
RBCs: Red Blood Cells
SCD: Sickle Cell Disease
SR: Systematic Review
VOEs: Vaso-occlusive Episodes/Events
